# Impact of Inaccurate Documentation of Sampling and Infusion Time in Model-Informed Precision Dosing

**DOI:** 10.3389/fphar.2020.00172

**Published:** 2020-03-03

**Authors:** Dzenefa Alihodzic, Astrid Broeker, Michael Baehr, Stefan Kluge, Claudia Langebrake, Sebastian Georg Wicha

**Affiliations:** ^1^ Department of Hospital Pharmacy, University Medical Center Hamburg-Eppendorf, Hamburg, Germany; ^2^ Department of Clinical Pharmacy, Institute of Pharmacy, University of Hamburg, Hamburg, Germany; ^3^ Department of Intensive Care Medicine, University Medical Center Hamburg-Eppendorf, Hamburg, Germany; ^4^ Department of Stem Cell Transplantation, University Medical Center Hamburg-Eppendorf, Hamburg, Germany

**Keywords:** documentation, sampling time, infusion rate, uncertainty, precision dosing, therapeutic drug monitoring, meropenem, caspofungin

## Abstract

**Background:**

Routine clinical TDM data is often used to develop population pharmacokinetic (PK) models, which are applied in turn for model-informed precision dosing. The impact of uncertainty in documented sampling and infusion times in population PK modeling and model-informed precision dosing have not yet been systematically evaluated. The aim of this study was to investigate uncertain documentation of (i) sampling times and (ii) infusion rate exemplified with two anti-infectives.

**Methods:**

A stochastic simulation and estimation study was performed in NONMEM^®^ using previously published population PK models of meropenem and caspofungin. Uncertainties, i.e. deviation between accurate and planned sampling and infusion times (standard deviation (SD) ± 5 min to ± 30 min) were added randomly in R before carrying out the simulation step. The estimation step was then performed with the accurate or planned times (replacing real time points by scheduled study values). Relative bias (rBias) and root mean squared error (rRMSE) were calculated to determine accuracy and precision of the primary and secondary PK parameters on the population and individual level. The accurate and the misspecified (using planned sampling times) model were used for Bayesian forecasting of meropenem to assess the impact on PK/PD target calculations relevant to dosing decisions.

**Results:**

On the population level, the estimates of the proportional residual error (prop.-err.) and the interindividual variability (IIV) on the central volume of distribution (V1) were most affected by erroneous records in the sampling and infusion time (e.g. rBias of prop.-err.: 75.5% vs. 183% (meropenem) and 10.1% vs. 109% (caspofungin) for ± 5 vs. ± 30 min, respectively). On the individual level, the rBias of the planned scenario for the typical values V1, Q and V2 increased with increasing uncertainty in time, while CL, AUC and elimination half-life were least affected. Meropenem as a short half-life drug (~1 h) was more affected than caspofungin (~ 9–11 h). The misspecified model provided biased PK/PD target information (e.g. falsely overestimated time above MIC (T > MIC) when true T > MIC was <0.4 and thus patients at risk of undertreatment), while the accurate model gave precise estimates of the indices across all simulated patients.

**Conclusions:**

Even 5-minute-uncertainties caused bias and significant imprecision of primary population and individual PK parameters. Thus, our results underline the importance of accurate documentation of time.

## Introduction

Routine clinical therapeutic drug monitoring (TDM) data is often used to develop population pharmacokinetic (PK) models, in particular for special patient populations (Broeker et al., 2018). Apart from developing dosing recommendations in these populations, PK models derived from clinical routine data are used for model-informed precision dosing to calculate the optimal dose in combination with individually measured patient characteristics (Wicha et al., 2015; Broeker et al., 2019).

In a daily clinical setting, it is often the case, that the planned sampling times are recorded rather than actual times, without being fully aware of the exact implications this may have. Indeed, uncertainties of a few minutes are considered as a minor deviation and are deliberately accepted. Previously Van der Meer et al. analyzed the influence of erroneous patient records on population pharmacokinetic modeling and individual Bayesian estimation and drew attention to a more conscious documentation of sampling times (Van Der Meer et al., 2012). Germovsek et al. (2016) reveal differences in the accuracy and precision of pharmacokinetic models based on different data bases. Their model was least biased and most precise despite the fact that other models had bigger datasets. Consequently, other factors like the documentation of the clinical data, which has not been carried out precisely enough in the TDM process, could have had an impact. Charpiat et al. (1994) also documented, that thoughtful data collection and documentation by trained clinical personnel is an essential part of therapeutic drug monitoring and necessary for predicting future serum concentrations with Bayesian fitted pharmacokinetic models. Yet, the size of the error in relation to the estimated pharmacokinetic parameters and the impact on different drugs and their properties has not been systematically explored.

Similarly, the documentation of uncertainties in the infusion rate is usually not done properly. The infusion rate of intravenously administered anti-infectives is often controlled *via* the dropping speed by adjusting a clamp, which is known to be inaccurate (Crass and Vance, 1985) instead of using an infusion pump that allows the exact adjustment of the infusion time. Hence, in clinical practice, it is difficult to document the exact infusion duration on a minute-basis, so the planned infusion times are usually documented according to the specifications of the product characteristics, without making sure that the infusion rate was truly adhered to. Unfortunately, this suggests that in the most cases this uncertainty remains undocumented.

The aim of this study was to investigate the consequences of uncertain documentation of (i) sampling times and (ii) infusion rate exemplified with two pharmacokinetically different drugs. This is intended, to clarify the requirement for accurate documentation when erroneous data is used for population pharmacokinetic modeling as well as model-informed precision dosing.

## Materials and Methods

### Selection of the Study Drugs

To compare the effects of uncertainty in documentation that may differ depending on the drug and its properties two different anti-infectives were chosen as examples. The pharmacokinetic profile of meropenem served as an example for a hydrophilic drug with an elimination half-life of about 1 hour. The pharmacokinetic profile of caspofungin served as an example for a lipophilic drug, with a higher terminal elimination half-life of 9–11 h.

### Study Design and Dataset Generation in ‘R’

For both drugs, a simulation dataset including 100 virtual patients was generated in R 3.6.1. The simulated dosing regimen for meropenem was 1,000 mg every 8 h with an infusion rate of 30 min (Medicines.org.uk, 2015), while in the simulated dataset of caspofungin an initial dose of 70 mg followed by a maintenance dose of 50 mg every 24 h infused over a 1-hour period was mimicked (Medicines.org.uk, 2017).

A rich sampling design was chosen, containing 10 sampling time points for meropenem measured in the first and third dosing occasion, and 17 sampling time points from 4 dosing intervals for caspofungin, respectively. The sampling times were optimized iteratively in simulation and estimation studies, whereby it was aimed to assure that the sampling design was itself unbiased. Sampling times were eventually set to 0.1, 0.7, 1.6, 4.1, 7.0, 15.0, 16.1, 16.7, 17.6, 20.1 h for meropenem and 0.5, 0.7, 12.0, 18.0, 23.5, 24.5, 30.0, 36.0, 47.5, 48.5, 54.0, 60.0, 71.5, 72.5, 78.0, 84.0, 95.5 h for caspofungin, resulting in unbiased sampling designs for both drugs. The sampling times were motivated by D-optimal sampling designs calculated in the TDMx software (www.TDMx.eu), and refined in stochastic simulation and estimation studies until an unbiased sampling design was established.

In order to better reproduce TDM data, a comparatively sparse sampling design was created. Thus, for every patient two sampling time points from the rich sampling design were randomly allocated using R 3.6.1.

### Population Pharmacokinetic Models

The pharmacokinetic models used for the study were two previously published 2-compartment population PK models of meropenem (Li et al., 2006) and caspofungin (Würthwein et al., 2013). The patient collective used for the meropenem model comprised of 79 hospitalized patients suffering from different types of infection. Similarly, for the caspofungin model data from 46 hospitalized patients suffering from an invasive aspergillosis were used. The most prominent difference between the two models was the presence of the inter-occasion variability (IOV), i.e. the variability that occurs in an individual patient between dosing intervals, in the model for caspofungin, which was integrated on the clearance. Given that 4 dosing intervals were studied, it was assured that IOV could be estimated for caspofungin. For both drugs, no covariates were included to simplify the workflow and focus on the study question. The models were encoded in NONMEM^®^ 7.4.3 and the analytical solution (ADVAN3) was used. FOCE-I (first order conditional estimate with interaction) was used for parameter estimation.

### Simulation and Estimation in NONMEM^®^

For each drug, a stochastic simulation and estimation study was performed in NONMEM^®^ 7.4.3 (ICON, Gaithersburg, MD, USA) (**Supplementary Figure S1**).

First, uncertainties, which means the deviation between accurate and planned sampling and infusion times, were added randomly using R 3.6.1 before carrying out the simulation step. These uncertainties for sampling and infusion times were given as the standard deviation (SD) and ranged from ± 5 min up to ± 30 min. Therefore the “rnorm”-function was used in R 3.6.1, which generates a vector of normally distributed random numbers. For sampling times, negative time points have been excluded and re-sampled due to clinical irrelevance corresponding to data below the limit of quantification in a real clinical scenario as they were sampled before the first dose was given. For infusion times the absolute value was used; if the infusion time was zero, a bolus injection was simulated.

Second, 1,000 clinical trial simulations including the uncertainty in sampling time or infusion rate were simulated for each drug. The simulated sampling and infusion times corresponded to the accurate times.

Subsequently, the estimation step was performed with the (i) accurate or (ii) planned times by replacing the accurate time points by scheduled study values. For the accurate scenario, the correctly documented time and settings were used for simulation. For the planned scenario, only the planned, wrongly documented time points were used.

### Impact on Estimated Pharmacokinetic Parameters on Population and Individual Level

For evaluation of the results, we compared the estimated PK parameters to the simulated (true) parameters. The relative bias (rBias) and the relative root mean squared error (rRMSE) were calculated to determine accuracy and precision, respectively, of the PK parameters:

$$\mathrm{rBias}\left[\%\right]=\frac{1}{N}\overset{i}{\underset{1}{\sum }}\frac{{\mathrm{estimated}}_{i}-{\mathrm{true}}_{i}}{{\mathrm{true}}_{i}}\times 100$$

$$\mathrm{rRMSE}\left[\%\right]=\sqrt{\frac{1}{N}\overset{i}{\underset{1}{\sum }}\frac{{\left({\mathrm{estimated}}_{i}-{\mathrm{true}}_{i}\right)}^{2}}{{{\mathrm{true}}_{i}}^{2}}\times 100}$$

where N is the number of simulated virtual trials.

rBias and rRMSE were evaluated on the population as well as on the individual (patient) level to finally compare the effect of uncertainty on correctly or incorrectly documented times.

### Calculation of the Time Above Minimum Inhibitory Concentration (T > MIC) for Meropenem, the Area Under the Curve (AUC_mod_/AUC_NCA_) for Caspofungin and the Half-Lives for Both Drugs

For meropenem we evaluated (i) how uncertain sampling affected the determined T > MIC, and (ii) the impact of using a pharmacometric model that was developed based upon planned times (uncertainty ± 30 min) rather than accurately documented times in Bayesian forecasting. To mimic a typical case for beta-lactam TDM, where dose optimization would have a role to ascertain PK/PD target attainment, a dose of 1500 mg q 8 h was administered as 1 h infusion and TDM sampling was performed at 2 and 7 h post dose. A MIC of 4 mg/L was assumed. The PK/PD target relevant for meropenem, i.e. the estimated time above the minimal inhibitory concentration (T > MIC) was assessed and compared to the true T > MIC. A total of 10,000 virtual patients were explored.

To quantify how inaccuracies in sampling times and infusion rates affect the secondary pharmacokinetic parameters of caspofungin, the AUC and the elimination half-lives were calculated. AUC is defined as the area under the concentration-time curve from dosing (time 0) to the time of the last measured concentration. The model based AUC approach (AUC_mod_) was compared to the estimated AUC using the non-compartmental (NCA) approach (AUC_NCA_), which in turn uses the trapezoidal rule to calculate the AUC. The AUC calculation was done for caspofungin within the first occasion and required an adjustment of the sampling times. Three sampling times were added at 1.0, 4.0 and 8.0 to enable a precise calculation of the AUC using the NCA approach.

The elimination half-life (Toutain and Bousquet-Melou, 2004) for each drug was calculated using the primary pharmacokinetic parameters clearance (CL), central volume of distribution (V1), distribution clearance (Q) and peripheral volume of distribution (V2).

## Results

### Sampling Time on Population Level

#### Meropenem

**Figure 1:**
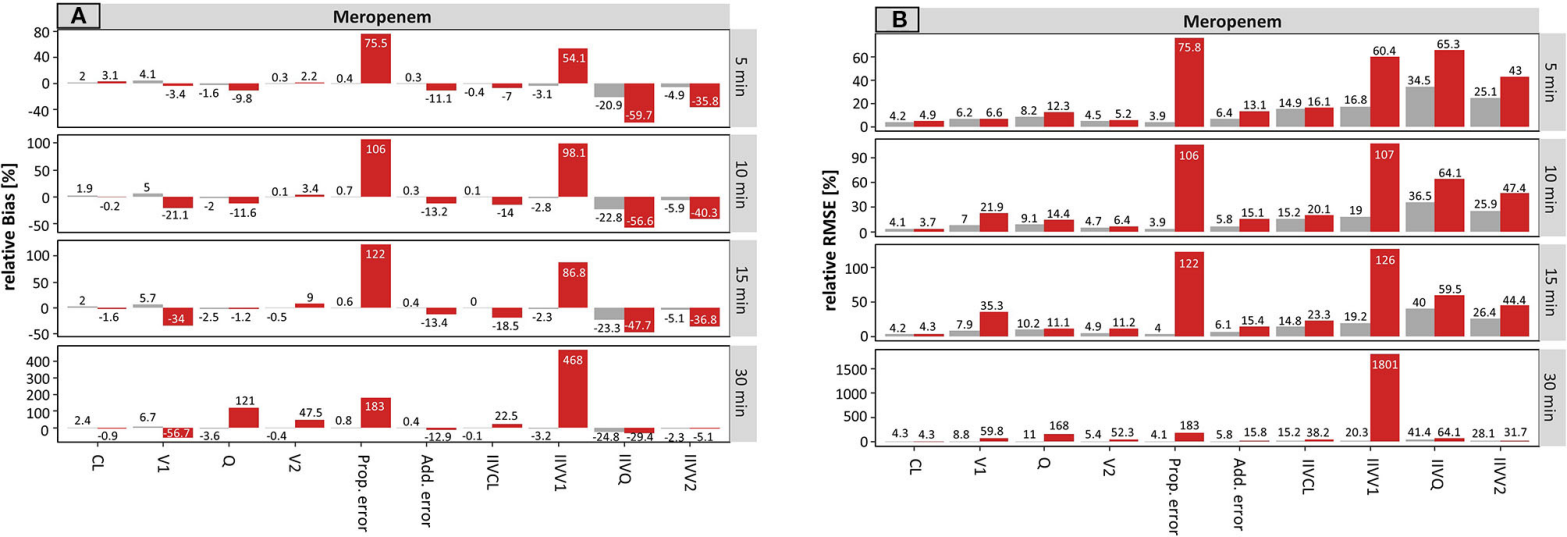
Accuracy (rBias, relative Bias) **(A)** and precision (rRMSE, relative root mean squared error) **(B)** for meropenem population pharmacokinetic parameters by uncertainty in sampling time (± 5 min to ± 30 min on standard deviation (SD) scale) using the accurate (grey) or planned (red) sampling times. Clearance (CL), central volume of distribution (V1), intercompartmental clearance (Q) and peripheral volume of distribution (V2), and the variability parameters on intraindividual (Prop./Add. error) and interindividual level (IIVs), respectively. The different scale size for each chart should be noted.

On the population level, the accuracy of the estimated parameters of meropenem with accurate and planned sampling times are presented in **Figure 1A**.

For the typical PK parameters, higher uncertainty in sampling times particularly affected the estimation of distribution parameters, i.e. the central (V1) and peripheral volume of distribution (V2) as well as the distribution clearance (Q). For example, the rBias for the central volume of distribution (V1) at an uncertainty of ± 5 min or ± 30 min in sampling time was −3.4 or −56.7% (planned) vs. 4.1 or 6.7% (accurate). Clearance was not affected. The scenarios where accurate documentation was used provided similar accuracy across all tested uncertainty times (5–30 min).

Stronger effects of undocumented sampling uncertainty were found in the estimates of the parameter variabilities. Even small undocumented uncertainty of ± 5 min in sampling time, increased the rBias of the estimated proportional residual error from 0.4 vs. 75.5% (accurate vs. planned, respectively), which increased further for ±30 min uncertainty (0.8% vs. 183%, accurate vs. planned, respectively). Moreover, the rBias in relation to the IIV on V1 was inflated at 5 min uncertainty (−3.1 vs. 54.1%, accurate vs. planned, respectively) and reached 468% (planned, −3.2% accurate) at an uncertainty of ± 30 min.

The precision of the estimated parameters (rRMSE) was also adversely affected by undocumented uncertainty in sampling time (**Figure 1B**). Again, the proportional error and the IIV on V1 were most affected by incorrect documented uncertainties. An increase from 3.9 to 75.8% (± 5 min) and from 4.1 to 183% (± 30 min) has been recorded for the proportional error. However, IIV on V1 was even more affected with values ranging from 60.4% (± 5 min) to 1801% (± 30 min).

#### Caspofungin

**Figure 2:**
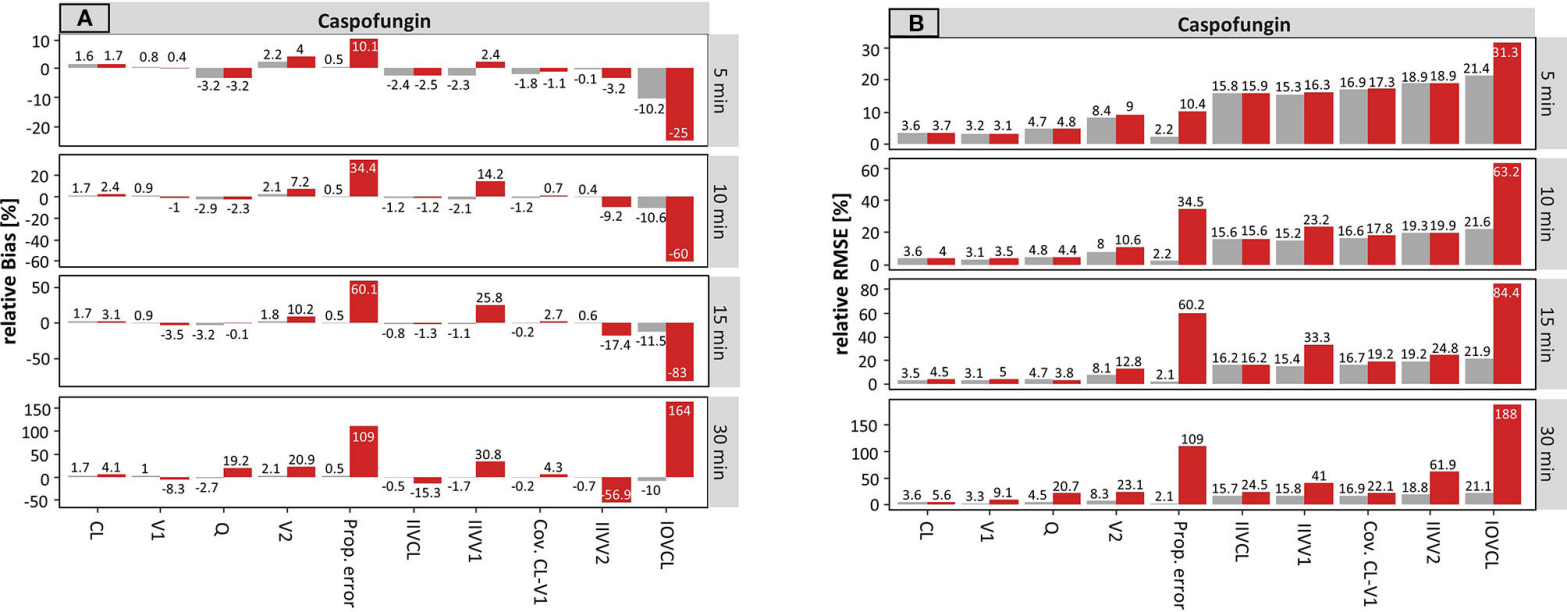
Accuracy (rBias) **(A)** and precision (rRMSE) **(B)** for caspofungin population pharmacokinetic parameters by uncertainty in sampling time (± 5 min to ± 30 min on SD scale) using the accurate (grey) or planned (red) sampling times. Clearance (CL), central volume of distribution (V1), intercompartmental clearance (Q) and peripheral volume of distribution (V2), and the variability parameters on intraindividual (Prop./Add. error) and interindividual level (IIVs), respectively. IOV: Inter-occasion variability. The different scale size for each chart should be noted.

The results for caspofungin concerning the sampling time are summarized in **Figure 2**. Similar effects can be observed as compared to meropenem, but these were less pronounced. The rBias (**Figure 2A**) of the planned times regarding the typical parameters was in a similar range as the accurate values, suggesting that the typical PK parameters were only slightly affected by incorrect documentation. The only parameter in which a significant effect with higher uncertainty was noticeable was V2 (e.g. for ± 30 min: 2.1 vs. 20.9%, accurate vs. planned, respectively).

The rBias for the proportional error increased with growing uncertainty. An increase from 0.5 to 10.1% (± 5 min) and from 0.5 to 109% (± 30 min) was recorded in this case. Similar effects were seen for the IIV on V1 and V2.

Compared to meropenem, a substantial difference is the presence of IOV. On the population level, the estimate of IOV was much affected by erroneous records in the sampling time. Even at an uncertainty of ± 5 min the rBias doubled from −10.2 to −25% in the sampling time. While the values of the IOV were underpredicted at smaller uncertainties, with higher uncertainties of e.g. ± 30 min the IOV was largely overestimated (164%).

**Figure 2B** depicts the rRMSE for caspofungin on the population level. In the same way, the proportional error and the IOV on CL were most affected by incorrect documented uncertainties. This is illustrated by an increase from 2.2 to 10.4% (± 5 min) and from 2.1 to 109% (± 30 min) for the proportional error. Whereas, values from 31.3% (± 5 min) to 188% (± 30 min) were obtained for the IOV considering the planned times. Taken together, undocumented uncertainty also affected the precision, similarly to the accuracy.

### Sampling Time on Individual Level

#### Meropenem

**Figure 3:**
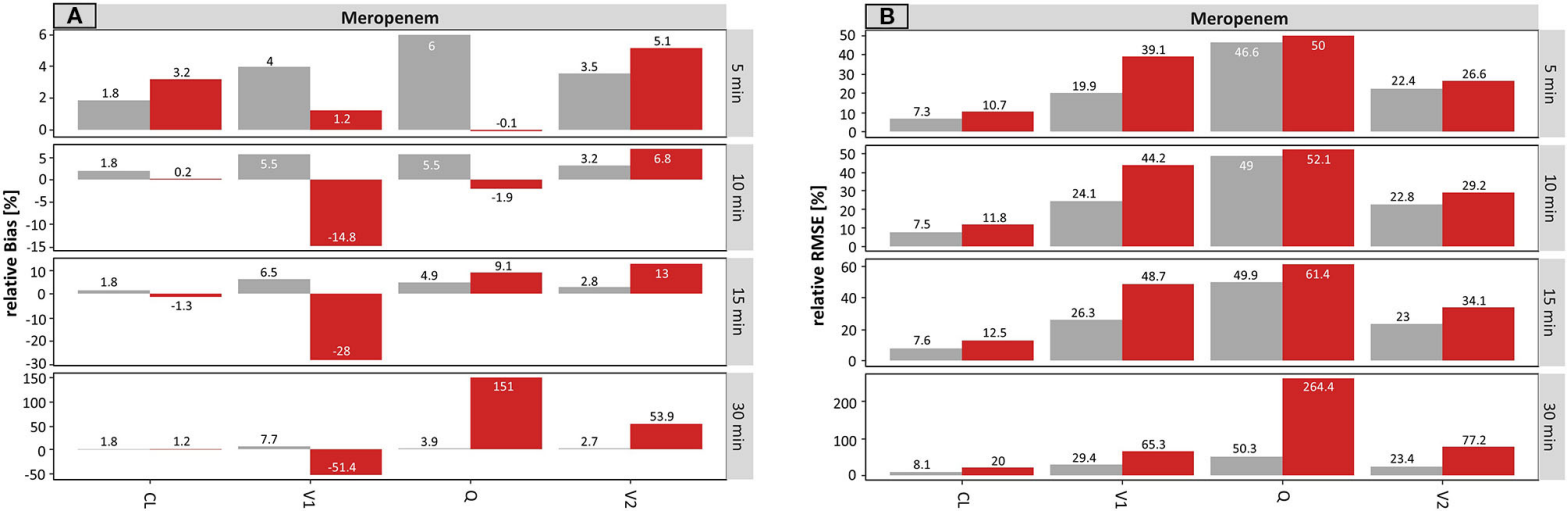
Accuracy (rBias) **(A)** and precision (rRMSE) **(B)** for meropenem individual pharmacokinetic parameters by uncertainty in sampling time (± 5 min to ± 30 min on SD scale) using the accurate (grey) or planned (red) sampling times. The different scale size for each chart should be noted. For an explanation of the abbreviations, see **Figure 1**.

On the individual level, the accuracy for the estimated individual parameters of meropenem with accurate and planned sampling time are presented in **Figure 3A**. The higher the uncertainty time, the higher the rBias of the planned scenario for the typical values V1, Q, and V2, while CL again apparently remained unaffected. While V1 was increasingly underestimated with rising uncertainty, Q and V2 were overestimated. Using incorrect documented (planned) sampling times, a rBias of −51.4% (V1) and 53.9% (V2), as well as a rBias of 151% (Q) for an uncertainty of ± 30 min were found, respectively.

**Figure 3B** presented the rRMSE of the same scenario for meropenem on the individual level. The initially small differences between accurate and planned times at an uncertainty of ± 5 min were rising with increasing uncertainty. Nevertheless, the impact of uncertainties was less pronounced, as compared to the results on the population level.

#### Caspofungin

**Figure 4:**
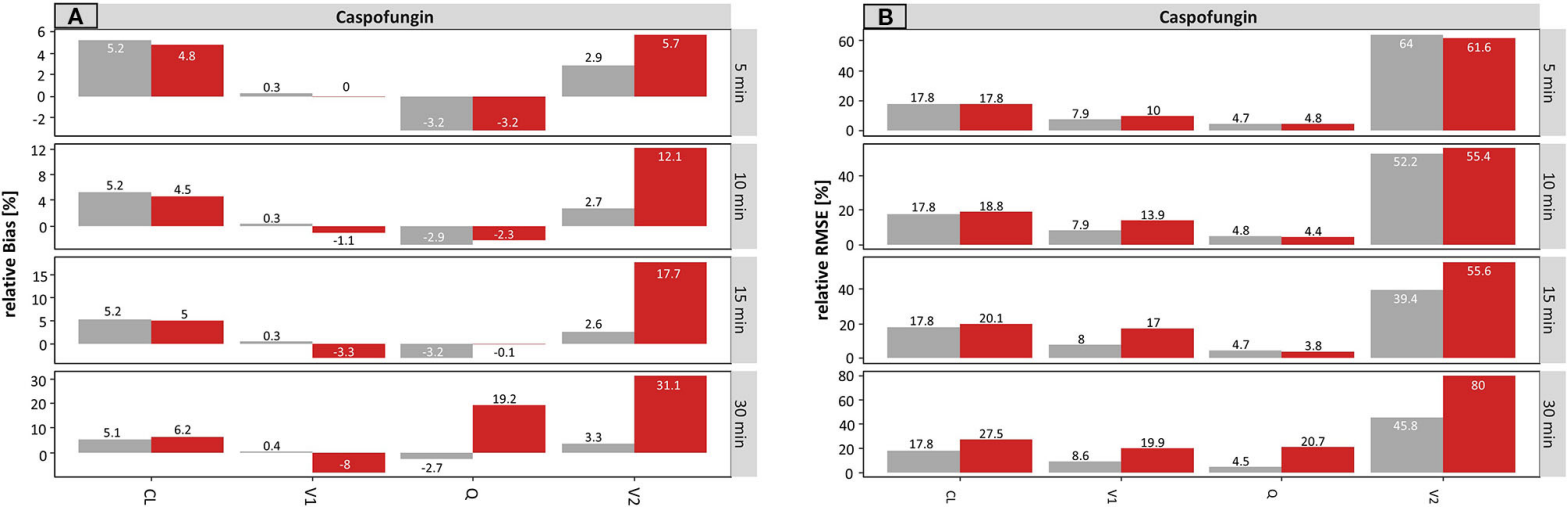
Accuracy (rBias) **(A)** and precision (rRMSE) **(B)** for caspofungin individual pharmacokinetic parameters by uncertainty in sampling time (± 5 min to ± 30 min on SD scale) using the accurate (grey) or planned (red) sampling times. The different scale size for each chart should be noted. For an explanation of the abbreviations, see **Figure 2**.

The results for caspofungin concerning the sampling time on the individual level are summarized in **Figure 4**. A minimal effect on V1 and Q was recognized even at a high uncertainty of ± 30 min. The only visible effect was seen on V2 (e.g. for ± 30 min: 3.3 vs. 31.1%, accurate vs. planned, respectively).

**Figure 4B** depicted the rRMSE for caspofungin on the individual level. The same tendency for the results was obtained as for rBias.

### Infusion Rate on Population Level

#### Meropenem

**Figure 5:**
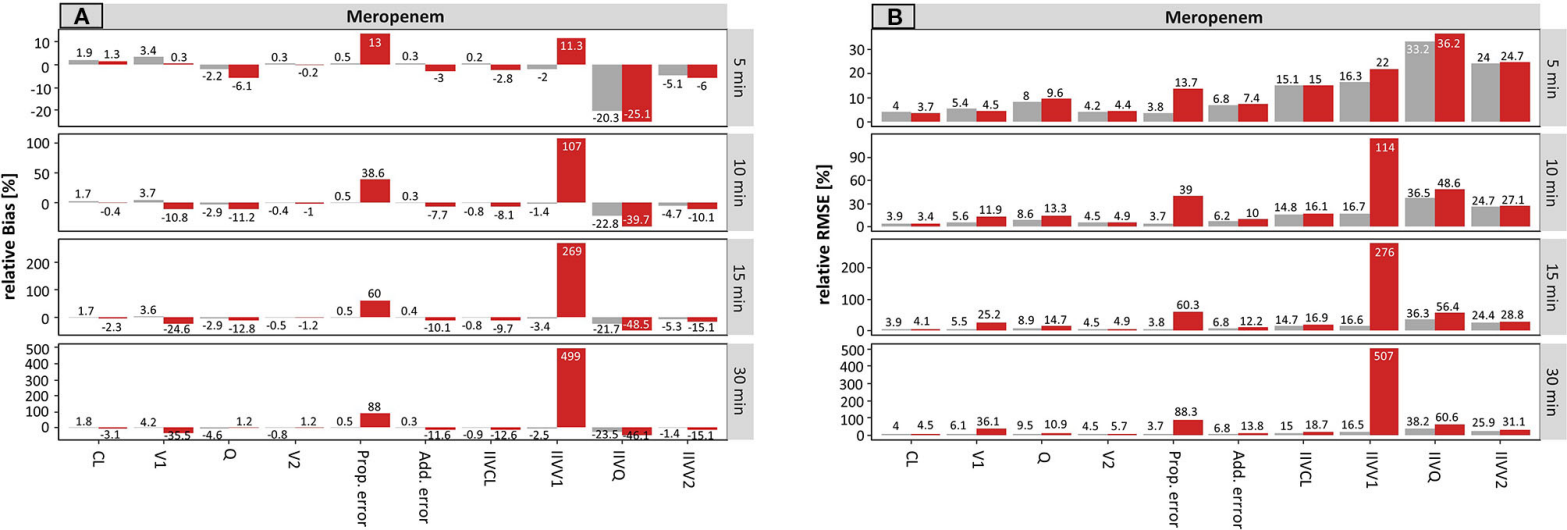
Accuracy (rBias) **(A)** and precision (rRMSE) **(B)** for meropenem population pharmacokinetic parameters by uncertainty in infusion rate (± 5 min to ± 30 min on SD scale) using the accurate (grey) or planned (red) infusion times. The different scale size for each chart should be noted. For an explanation of the abbreviations, see **Figure 1**.

On the population level, the results for meropenem concerning the infusion rate are presented in **Figure 5**.

For the typical PK parameters, V1 was mainly affected by incorrect documentation of the infusion rate. The higher the uncertainty time, the higher the resulting rBias of the planned scenario (**Figure 5A**), while the accurate scenario provided similar accuracy across all tested uncertainty times (5 – 30 min). With rising uncertainty, V1 was increasingly underestimated. This is illustrated by the rBias for V1 at an uncertainty of ± 5 min or ± 30 min in infusion rate: 0.3 or −35.5% (planned) vs. 3.4 or 4.2% (accurate).

Stronger effects were observed in the estimates of the parameter variabilities. A small undocumented uncertainty of ± 5 min in infusion rate, increased the rBias of the estimated proportional residual error from 0.5 to 13% (accurate to planned, respectively), which increased further for ± 30 min uncertainty (0.5 vs. 88%, accurate vs. planned, respectively). Moreover, the rBias in relation to the IIV on V1 was inflated at 5 min uncertainty (−2.0 vs. 11.3%, accurate vs. planned, respectively) and reached 499% (planned, −2.5% accurate) at an uncertainty of ± 30 min.

Compared to the results for the sampling time, a lower but still substantial impact of uncertainty in the infusion rate was noticed.

**Figure 5B** displays the precision (rRMSE) of the same scenario, for meropenem on the population level. Again, the proportional error and the IIV on V1 were most affected by incorrect documented uncertainties. An increase from 3.8 to 13.7% (± 5 min) and from 3.7 to 88.3% (± 30 min) was recorded for the proportional error. However, even more affected was the IIV on V1 with values ranging from 22.0% (± 5 min) to 507% (± 30 min) considering the planned times.

#### Caspofungin

**Figure 6:**
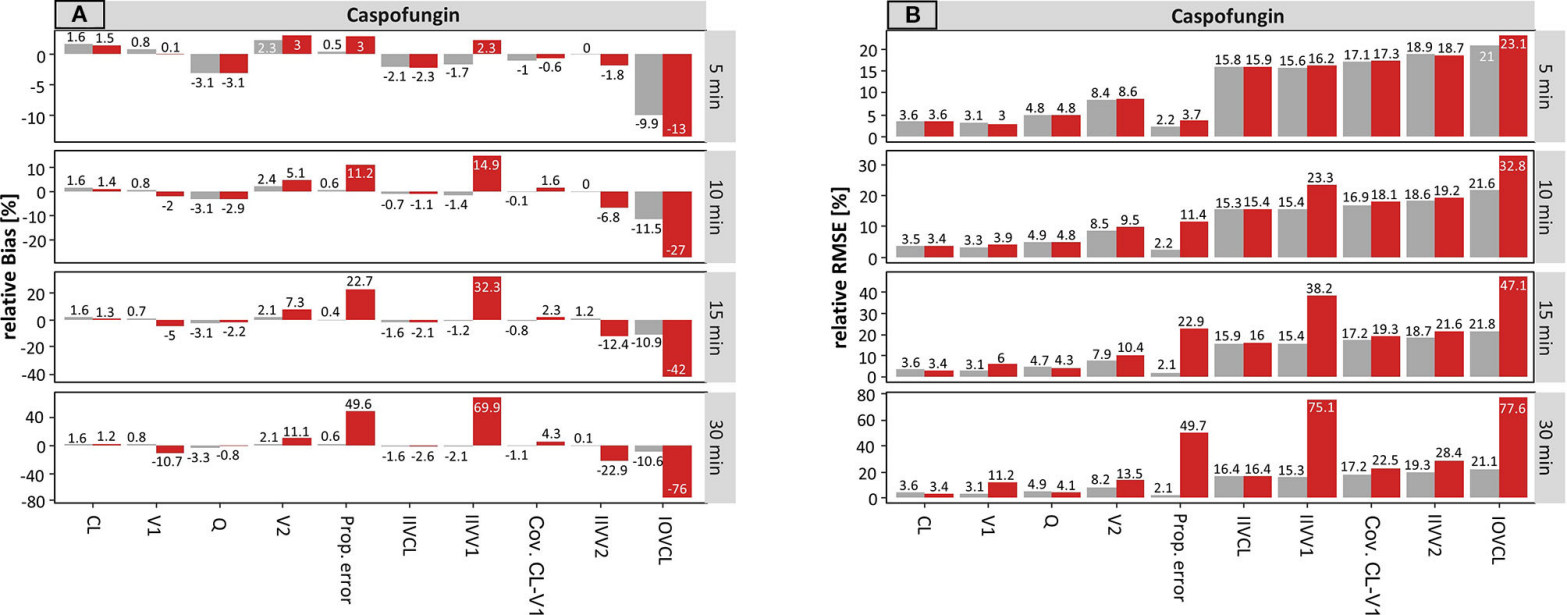
Accuracy (rBias) **(A)** and precision (rRMSE) **(B)** for caspofungin population pharmacokinetic parameters by uncertainty in infusion rate (± 5 min to ± 30 min on SD scale) using the accurate (grey) or planned (red) infusion times. The different scale size for each chart should be noted. For an explanation of the abbreviations, see **Figure 2**.

The results for caspofungin concerning the infusion rate are summarized in **Figure 6**. Similar effects were observed compared to meropenem, but these were less pronounced. The rBias (**Figure 6A**) of the planned times regarding the typical parameters was in a similar range as the accurate values, suggesting that the typical PK parameters were only slightly affected by incorrect documentation. A minimal effect can be seen on V1 and V2 (e.g. for ± 30 min: −10.7 and 11.1% concerning planned times, respectively).

The rBias for the proportional error increased with increasing uncertainty. An increase from 0.5 to 3.0% (± 5 min) and from 0.6 to 49.6% (± 30 min) was recorded in this case. Similar effects were seen for the IIV on V1 and V2, while the effect on IIV–V1 was much more pronounced.

Again, the estimate of IOV on CL was influenced the most by erroneous records in the infusion rate. The values of the IOV increased with increasing uncertainty and were underestimated throughout. For instance, the IOV at an uncertainty of ± 30 min was −10.6 vs. −76% (accurate vs. planned, respectively).

**Figure 6B** depicts the rRMSE for caspofungin on the population level for the infusion rate. In the same way, the proportional error, the IIV on V1 and the IOV on CL were most affected by incorrect documented uncertainties. This effect was most apparent at a high uncertainty of ± 30 min.

### Infusion Rate on Individual Level

#### Meropenem

**Figure 7:**
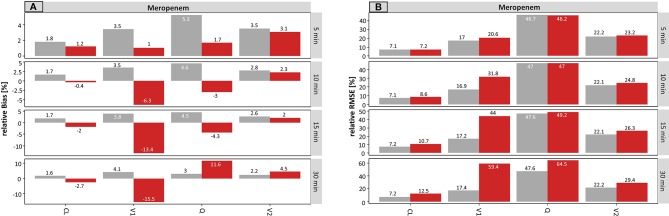
Accuracy (rBias) **(A)** and precision (rRMSE) **(B)** for meropenem individual pharmacokinetic parameters by uncertainty in infusion rate (± 5 min to ± 30 min on SD scale) using the accurate (grey) or planned (red) infusion times. The different scale size for each chart should be noted. For an explanation of the abbreviations, see **Figure 1**.

On the individual level, the results for the infusion rate are presented in **Figure 7**. Only a minimal impact on V1 was notable. Here, a rBias of −15.5% and a rRMSE of 59.4% was obtained at an uncertainty of ± 30 min. An impact of uncertainties in the infusion rate on CL, Q, and V2 was missing.

#### Caspofungin

**Figure 8:**
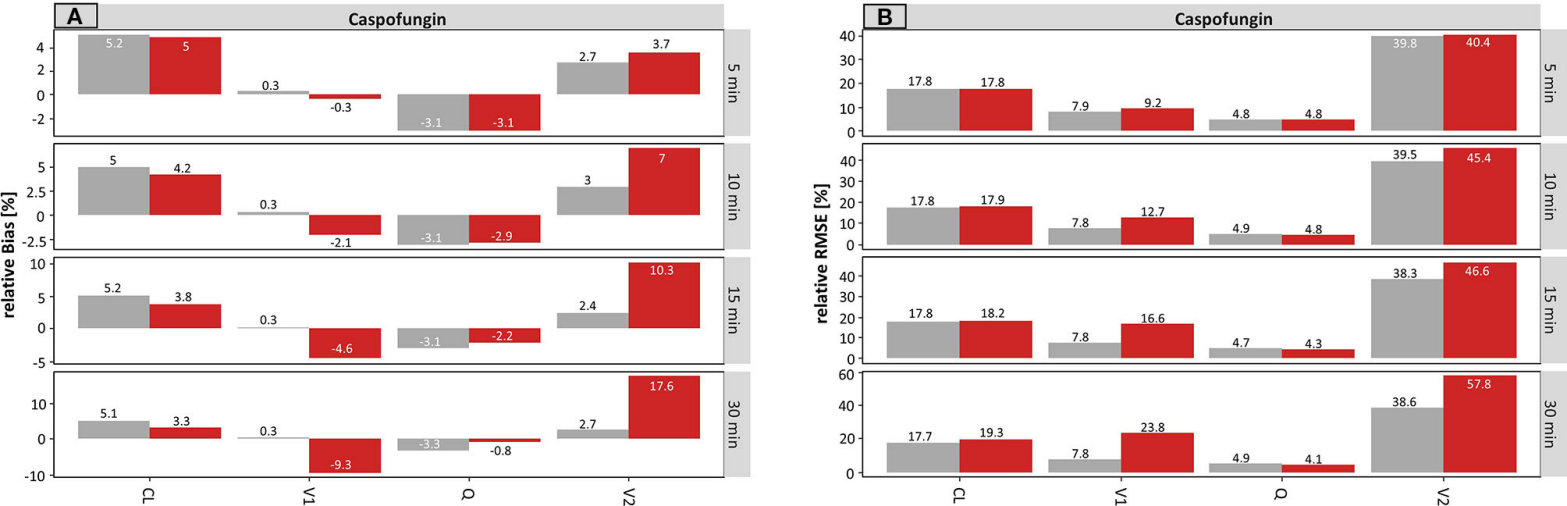
Accuracy (rBias) **(A)** and precision (rRMSE) **(B)** for caspofungin individual pharmacokinetic parameters by uncertainty in infusion rate (± 5 min to ± 30 min on SD scale) using the accurate (grey) or planned (red) infusion times. The different scale size for each chart should be noted. For an explanation of the abbreviations, see **Figure 2**.

Regarding caspofungin a minimal impact on V2 was registered. In particular, a rBias of 17.6% and a rRMSE of 57.8% was obtained at an uncertainty of ± 30 min (**Figure 8**).

### Impact of Sparse Sampling

The accuracy and the precison of the estimated parameters of meropenem and caspofungin with accurate and planned sampling times and infusion rates using a sparse sampling are presented in **Supplementary Figures S2–S9**. Compared to the respective results of the rich sampling, a similar pattern regarding the impact on the PK parameters was observed. Higher imprecision and inaccuracy occurred for each uncertainty time. Again, stronger effects of undocumented sampling uncertainty were found in the estimates of the parameter variabilities. Taken together, the proportional error and the IIV on V1 were most affected.

For meropenem a significant impact on Q (rBias − rich vs. sparse at ± 30 min sampling time: 121 vs. 214.9%) could be observed, while the impact on the IIV on V1 (rBias − rich vs. sparse at ± 30 min sampling time: 468 vs. 187.9%) decreased. Similarly, for caspofungin an increase in the IIV on V1 (rBias − rich vs. sparse at ± 30 min sampling time: 30.8 vs. 327%) could be observed, while the impact on the IOV (rBias − rich vs. sparse at ± 30 min sampling time: 164 vs. 23.7%) decreased.

However, it should be emphasized, that with a smaller number of sampling time points, the values determined using accurate documentation were more biased, especially on an individual level. The rBias e.g. for V1 of meropenem was 8.1 to 11.4% (accurate values: ± 5 min to ± 30 min) and the corresponding rRMSE was 40.6 to 45.1% (accurate values: ± 5 min to ± 30 min), respectively.

### Using an Accurate and the Misspecified Model (Developed on Uncertainly Sampled Data) in Bayesian Forecasting Exemplified for Meropenem

**Figure 9:**
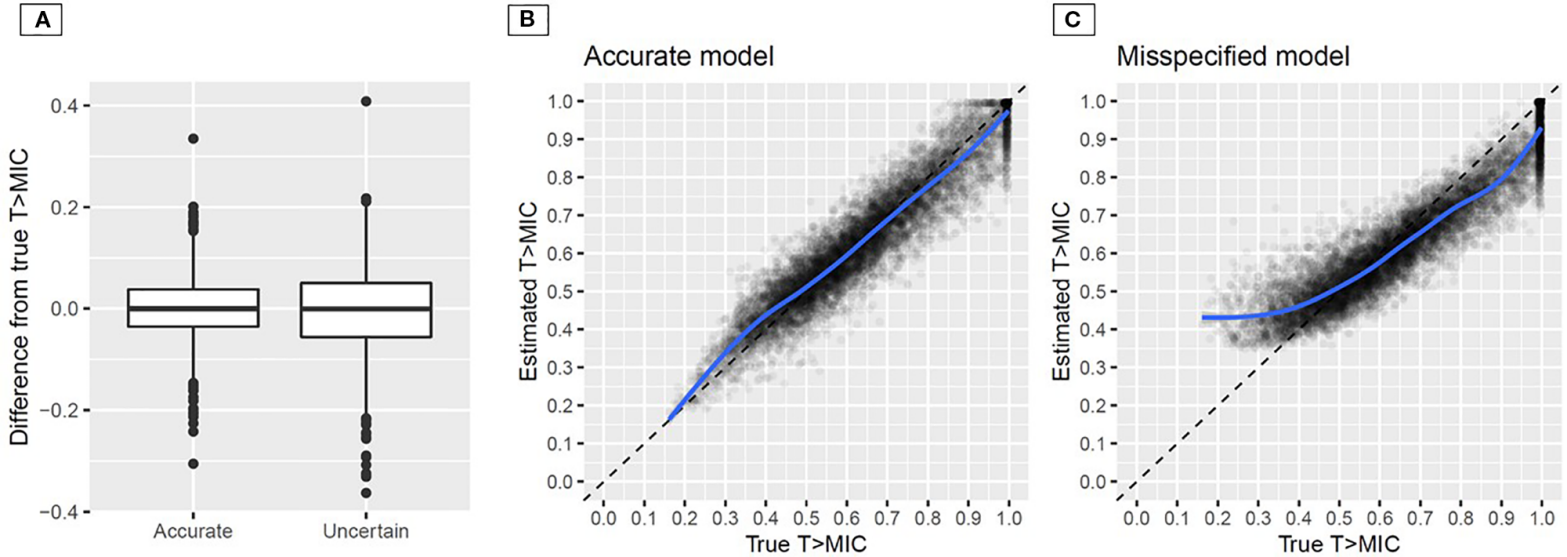
Difference between true and estimated T > MIC for accurate vs. uncertain documentation of sampling time using a pharmacometric model that was developed on accurately documented sampling time **(A)**; estimated vs. true time above minimum inhibitory concentration (MIC) for the accurate **(B)** and the misspecified **(C)** model (developed based on planned times) in Bayesian forecasting. The blue line indicates a smoothed conditional mean; Uncertainty in sampling time: ± 30 min (SD).

Uncertain documentation of sampling time (undocumented uncertainty, SD of ± 30 min) affected the accuracy of the determined T > MIC (**Figure 9A**). While there was no systematic bias observed, the number of outliers where T > MIC was determined with >0.1 difference occurred in 11 or 23% of the cases for accurate vs. uncertain documentation using planned times.

The second scenario, i.e. using a model that was developed based upon TDM data with planned times (undocumented uncertainty ± 30 min) or accurate times is presented in **Figure 9** for the example of meropenem. In **Figure 9B**, the case of the model that was developed on accurate documentation is presented. Over the full range the model adequately estimated T > MIC with 10.7% CV. Conversely, when the misspecified model was used (**Figure 9C**), T > MIC was underestimated at high T > MIC above 0.7 and overestimated for T > MIC below 0.5. In particular, the latter result is dangerous as adequate therapy with T > MIC ≥0.4 would be assumed while patients are not above the minimum PK/PD target of 0.4, but at risk of treatment failure. Notably, accurate sampling times were used for Bayesian forecasting in this example, but the effect originated from the misspecified model developed upon planned times.

### Model Based AUC_mod_ and the AUC Using the NCA (AUC_NCA_) Approach Exemplified for Caspofungin

**Supplementary Figure S10** displays accuracy and precision of the model based AUC_mod_ of caspofungin with accurate and planned sampling times and infusion rates, respectively, compared to the AUC using the NCA approach. The rBias (**Supplementary Figure S10A**) and the rRMSE (**Supplementary Figure S10B**) were in a similar range for the accurate and the planned scenario as well as for the model based and the NCA approach. Overall the precision and accuracy of the estimated AUC was only slightly affected by incorrect documentation (highest rBias: −3.43% for ± 15 for min planned sampling time, highest rRMSE: 11,63% for ± 30 min planned sampling time).

The only approach not affected by uncertainty on sampling time were the accurate values for AUC_mod_, where rBias for the sampling time ranged from -0.58% to -0.61% and rRMSE: from 4.81% to 4.92% across all studied uncertainty times.

### Impact on Estimated Half-Life

For meropenem, the rBias for the elimination half-life calculation concerning the sampling time is summarized in **Supplementary Figure S11A**. Overall, the rBias was below 4% in all presented cases. Subsequently, an impact of uncertainties of the sampling time on the elimination half-life of meropenem is not apparent.

**Supplementary Figure S11B** represents the effect on the precision concerning the elimination half-life on individual level. For example, the rRMSE at an uncertainty of ± 5 min or ± 30 min in sampling time was 19 or 60.8% (planned) vs. 17 or 18.4 % (accurate).

For caspofunign, similar results were obtained, presented in **Supplementary Figure S12**. The results concerning the infusion rate can be found in the **Supplementary Material** (**Figures S13** and **S14**).

## Discussion

The present study reveals considerable impact of inaccurate documentation of sampling and infusion times on the accuracy and precision of estimated population and individual parameters as well as adverse effects on PK/PD target attainment calculations in model-informed precision dosing.

Our data demonstrate that on an overall basis, the typical parameters are relatively robust for smaller imprecisions, especially related to clearance. The secondary PK parameters (AUC and elimination half-life) remain relatively unaffected, as well. The AUC is inversely proportional to the clearance of the drug and consequently reveals a similar behavior towards inaccurate documentation compared with the clearance. There is no significant difference, comparing the two different AUC calculation methods (model based vs. NCA). Nevertheless, it should be noticed that a total of 8 sampling times within a dosing interval were necessary to be able to exactly calculate the AUC_NCA_ using the trapezoidal method, whereas for the AUC_mod_ calculation two sampling times over different occasions are sufficient. The results for the AUC were only generated for caspofungin, since the PK/PD target of meropenem is not AUC-dependent.

However, some typical parameters, in particular distribution related parameters, were misspecified and more imprecisely estimated even for an uncertainty of 10 min in sampling time and 15 min in infusion rate compared to accurately documented data. This effect was more pronounced with higher uncertainty. Overall, the effect of uncertainty in documented time was more pronounced for the meropenem example, which can be explained by the shorter elimination half-life of the drug, where changes in the concentration-time profile occur more rapidly.

In contrast, the estimated variability components of the pharmacometric model were affected more substantially by uncertain documentation. In particular, the residual variability was strongly influenced – even for small uncertainties over all scenarios. In addition, we determined a high influence on the estimated IIV and IOV. For the IIV, accurate and precise estimations were not guaranteed, even for smaller uncertainty times of 5 or 10 min. For the IOV, the impact was even more pronounced leading first to under- and at high uncertainty to overestimation, if planned times were utilized. Hence, the validity of estimated IOV needs to be questioned in studies attempting to parameterize IOV from clinical routine data when small sampling errors occurred.

On the individual level, the differences between planned and accurate times were less marked. This might be due to the high sample size per patient. Only high uncertainties in sampling time lead to considerable, structural misspecification on the individual level.

A similar pattern regarding the impact on the PK parameters was observed using a sparse sampling design. Compared to the rich sampling, higher imprecisions and inaccuracy occurred and suggested a higher importance for accurate documentation of sampling and infusion times. For the sparse sampling design, two sampling time points from the rich sampling design were randomly allocated using R 3.6.1. This could be advantageous, since a lot of different sampling information is available for the parameter prediction. Furthermore, it can be assumed, that the use of a sparse sampling design without selecting the sampling time points randomly, would further increase on the one hand the potential impact of inaccurate documentation and on the other hand the need for higher awareness.

For the PK/PD analysis, for the example of meropenem, the number of cases where T > MIC was determined wrongly with a difference >0.1 increased from 11 to 23% when planned rather than accurate sampling times were used. More problematic, however, was the use of a pharmacometric model that was developed using concentration-time data assuming planned sampling times. Even when actual sampling times were used to estimate PK/PD target attainment, the example of meropenem (**Figure 9**) illustrates that wrong therapeutic conclusions might be drawn when such a model is used in model-informed precision dosing. The reasons for the biased estimation of time might be found in misspecified typical PK parameters and so called “Shrinkage” (Savic and Karlsson, 2009), where the model prediction of the posteriori Bayesian estimates shrinks to the population mean, here due to the inflated residual variance. The chosen example of meropenem is highly relevant given that beta-lactam TDM and dose individualization is increasingly performed in ICU patients and recommended by contemporary guidelines (Guilhaumou et al., 2019). A consequence might be that patients with low T > MIC >0.4 are falsely categorized as receiving adequate treatment given that the model would estimate T > MIC values of 0.4 and larger, whereas high T > MIC would be underestimated, putting patients “at risk” of a not required dose increase.

By examining two different drugs, we identified that the strength of influence of uncertainties might also depend on the pharmacokinetic properties of the used drug. The population PK parameters of meropenem have been more affected by inaccurate documentation, likely due to the shorter elimination half-life and the thus higher influence of uncertainty on the time axis on all distribution phases. Uncertainties in the sampling time displayed a stronger effect than uncertainty in documented infusion rate. Uncertainty in infusion rate influenced mainly the estimation of V1, while the sampling time affected all distribution parameters. However, the documentation of the infusion rate is a persistent problem in clinical practice. While erroneous records on sampling time have been discussed (Van Der Meer et al., 2012), the infusion rate has been neglected. Since not only recording but also adjusting of the correct infusion time is often disregarded in clinical practice, it might therefore have a greater impact on the accuracy of routine TDM data.

Van der Meer et al. also investigated the influence of uncertainties on the sampling time. Four crucial differences to our work shall be emphasized: In our work, we varied the uncertainties randomly, while Van der Meer et al. systematically generated the different errors with a fixed error magnitude of 2 h, which does not entirely mimic clinical practice. In addition, the dependence of the error from the magnitude of uncertain sampling time has not been studied, while our approach shows that already very small undocumented uncertainty can be impactful and the impact was investigated more granularly. Also, their study solely investigated the effect on the parameters of vancomycin. A comparison of different drugs has not taken place. Lastly, the influence of uncertainties on the infusion rate was not considered. Hence, our study complements the study of Van der Meer et al.

Finally, this work may change the way we view the role of clinical routine TDM data, which may be used in population PK modeling and finally for model-informed precision dosing. If routine data is used for this purpose, it is particularly important to clarify the need of accurate documentation to all professionals involved in the clinical conduct of TDM. The aspect of accurate documentation will also be of high importance to model-based precision dosing software that are embedded in electronic health record systems and use routine data in a continuous learning approach to improve the model’s predictive performance.

Nevertheless, some limitations of this study shall be discussed: The comparison between the meropenem and caspofungin results is not directly possible, as the scenarios differ in the number of sampling points per patient (10 vs. 17 sampling points, respectively). The number of sampling points is related to the parameter precision and in general more samples allow for more accurate and precise estimation. However, the different number of sampling time-points had explicit reasons: On the one hand, we aimed to study an unbiased design when no uncertainty was included to avoid misleading results and on the other hand, due to the presence of the IOV in the caspofungin model, the precise estimation of the IOV was ensured by sampling over four dosing occasions.

In addition, regardless of the medical question, no covariate-relationship was included in the selected models for meropenem and caspofungin. Hence, in future studies it might be interesting to explore the impact of uncertain documentation one estimated covariate relationships.

Moreover, the sampling schedule was optimized to be unbiased when accurate sampling was used. This optimization lead to sampling during the infusion phase, which is clinically challenging and not standard practice.

Furthermore, the impact of uncertainties in the documented administration time of different drugs was not included in the study, even though similar effects can be expected. The same applies for different patient or sample sizes. The investigation of further drug examples with different properties was not within in the scope of this study. However, a drug like tamoxifen (Klopp-Schulze et al., 2018), which has a long elimination half-life of typically 5 to 7 days and where the steady state levels are reached after 3 to 4 weeks, would be interesting to study as well.

In summary, the present study reveals the importance for accurate documentation of sampling and infusion times in population PK modeling and model-informed precision dosing. Erroneous records, even with small uncertainty in documented time considerably impact the accuracy and the precision of estimated PK parameters on the population and individual level. Our work offers insights that this situation becomes particularly critical, if routine clinical TDM data is used to develop population pharmacokinetic models, which are in turn used in model-informed precision dosing. In the worst-case, undocumented sampling or infusion time can erroneously lead to wrong clinical decisions in Bayesian forecasting. Our results provide a proof-of-principle demonstration that a correct documentation is vital for model-informed precision dosing and should deserve high attention.

## Data Availability Statement

The datasets generated for this study are available on request to the corresponding author.

## Author Contributions

DA, AB, and SW contributed to the conception, study design, pharmacometric analysis and interpretation of the data. MB, SK, and CL provided clinical input and interpretation of the data. DA and SW drafted the manuscript. AB, MB, SK, and CL revised the manuscript for intellectual content. DA, AB, MB, SK, CL, and SW approved the final version to be published. All authors agreed to be accountable for all aspects of the work.

## Funding

The present study was funded by institutional sources.

## Conflict of Interest

The authors declare that the research was conducted in the absence of any commercial or financial relationships that could be construed as a potential conflict of interest.
