# Supplementary material for: Impact of Inaccurate Documentation of Sampling and Infusion Time in Model-Informed Precision Dosing
Source: Front Pharmacol. 2020 Mar 3;11:172. doi: 10.3389/fphar.2020.00172 (PMC7063976; doi:10.3389/fphar.2020.00172)
Supplement: Supplementary file 1 [file DataSheet_1.docx]

Supplementary Material


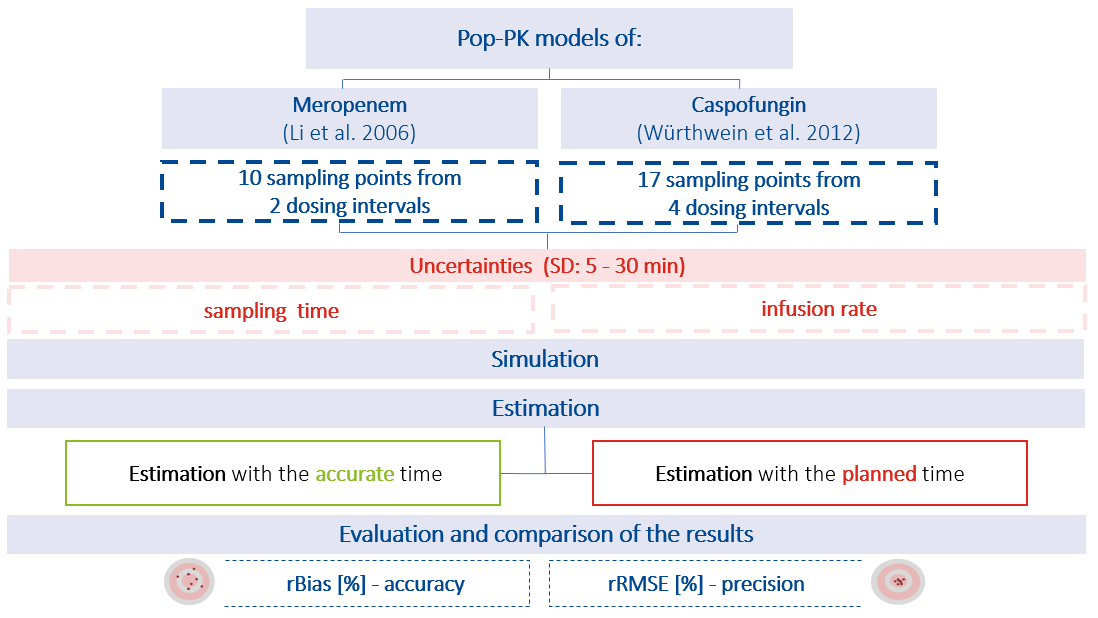


**Supplementary Figure S1.** Flow chart of the simulation and estimation study in NONMEM^®^/R. SD: standard deviation, rBias: relative Bias – measure for accuracy, rRMSE: relative root mean squared error – measure for precision.


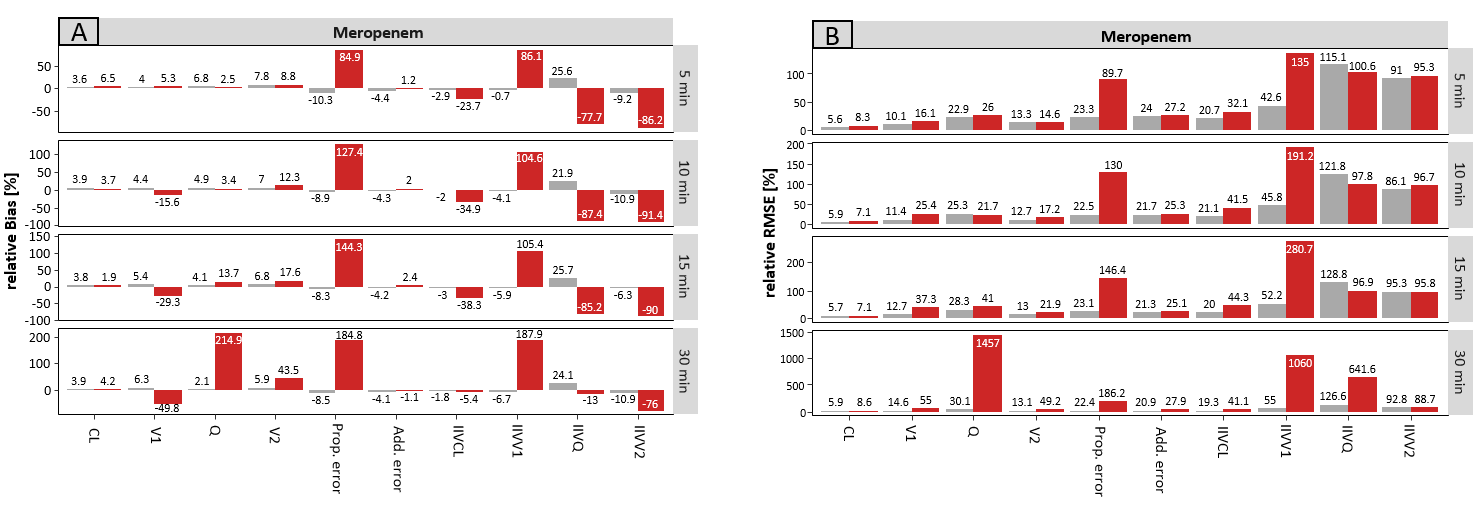


**Supplementary Figure S2.** Impact of sparse sampling. Accuracy (rBias, relative Bias) **(A)** and precision (rRMSE, relative root mean squared error) **(B)** for meropenem population pharmacokinetic parameters by uncertainty in sampling time (± 5 min to ± 30 min on standard deviation (SD) scale) using the accurate (grey) or planned (red) sampling times. Clearance (CL), central volume of distribution (V1), intercompartmental clearance (Q) and peripheral volume of distribution (V2), and the variability parameters on intraindividual (Prop./Add. error) and interindividual level (IIVs), respectively. The different scale size for each chart should be noted.


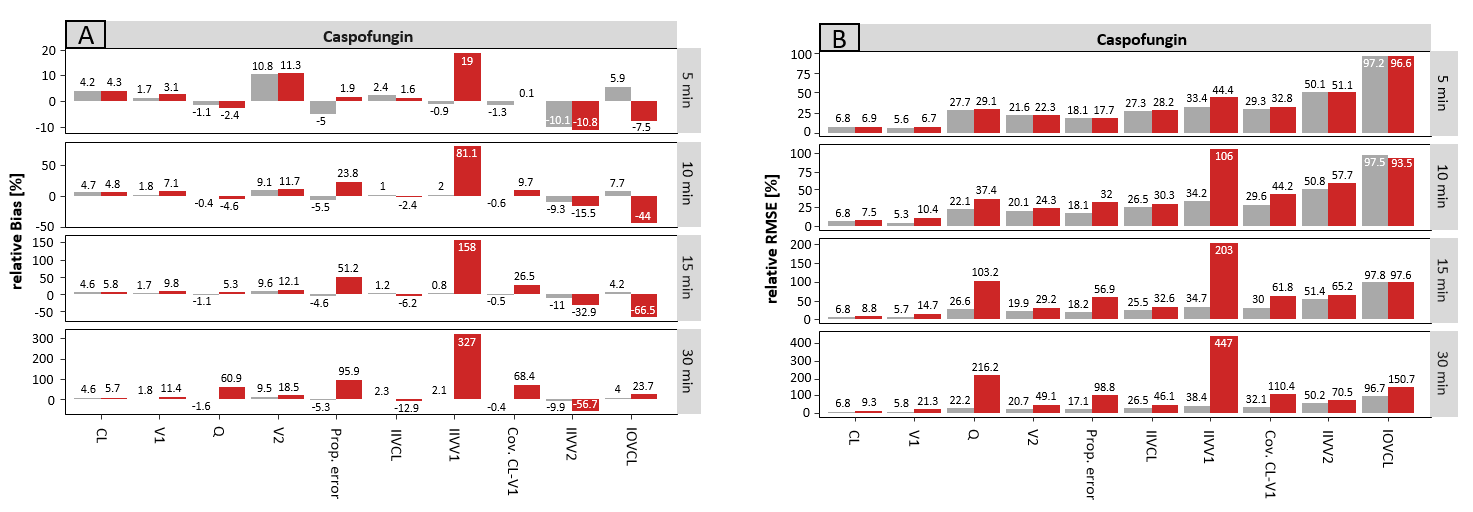


**Supplementary Figure S3.** Impact of sparse sampling. Accuracy (rBias) **(A)** and precision (rRMSE) **(B)** for caspofungin population pharmacokinetic parameters by uncertainty in sampling time (± 5 min to ± 30 min on SD scale) using the accurate (grey) or planned (red) sampling times. Clearance (CL), central volume of distribution (V1), intercompartmental clearance (Q) and peripheral volume of distribution (V2), and the variability parameters on intraindividual (Prop. error) and interindividual level (IIVs), respectively. Cov. CL-V1: off-diagonal covariance between CL and V1. IOV: Inter-occasion variability. The different scale size for each chart should be noted.


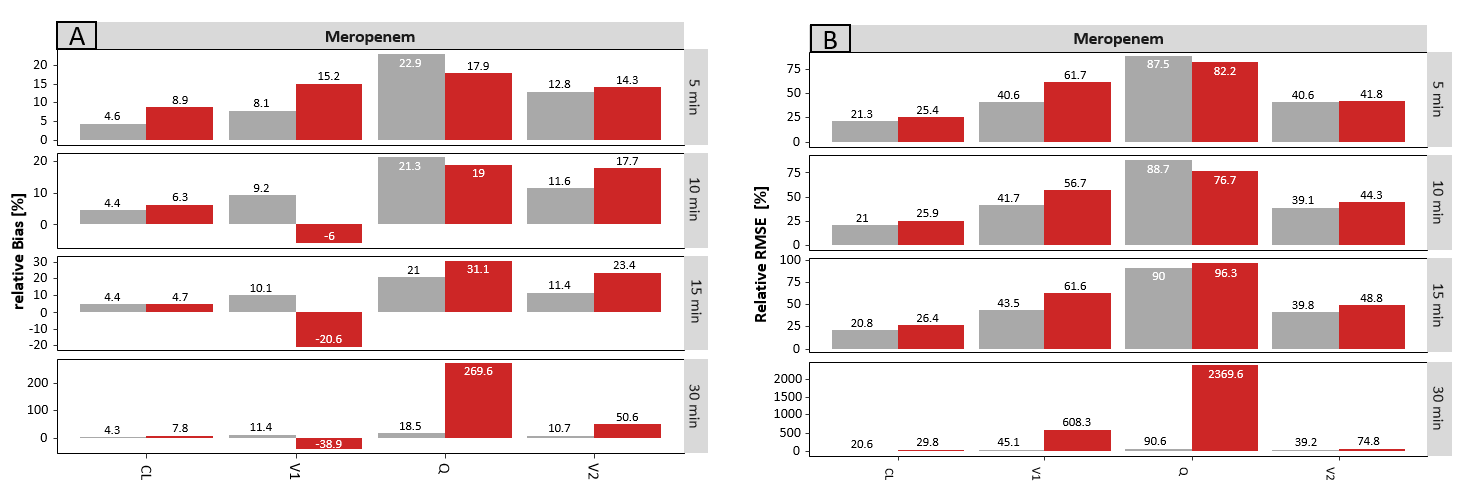


**Supplementary Figure S4.** Impact of sparse sampling. Accuracy (rBias) **(A)** and precision (rRMSE) **(B)** for meropenem individual pharmacokinetic parameters by uncertainty in sampling time (± 5 min to ± 30 min on SD scale) using the accurate (grey) or planned (red) sampling times. The different scale size for each chart should be noted. For an explanation of the abbreviations, see **Figure S2**.


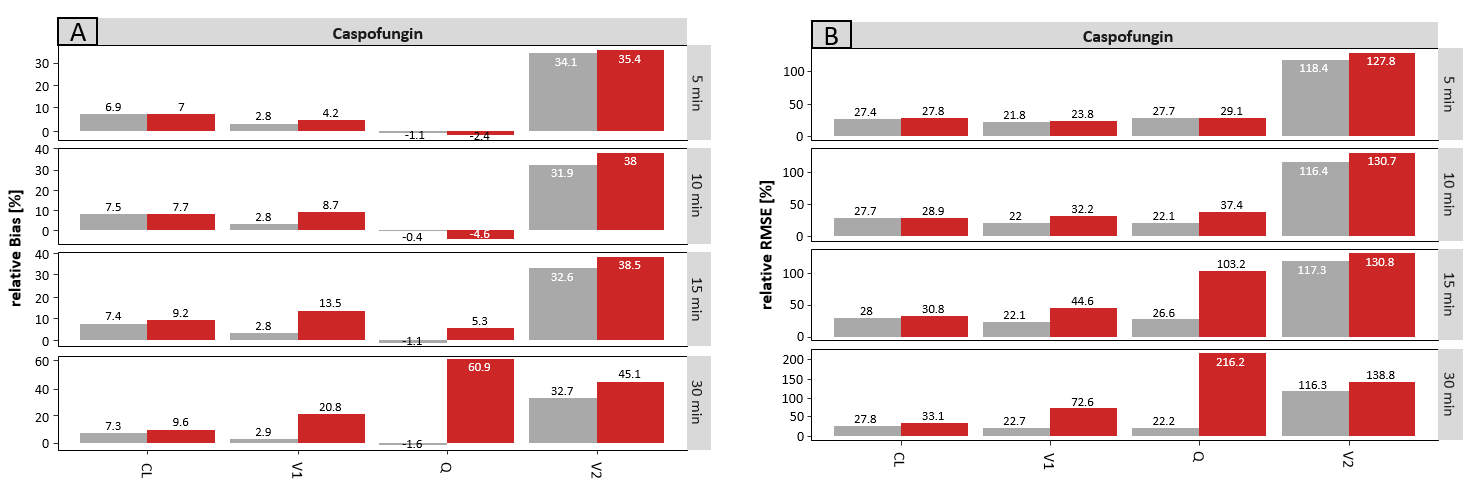


**Supplementary Figure S5.** Impact of sparse sampling. Accuracy (rBias) **(A)** and precision (rRMSE) **(B)** for caspofungin individual pharmacokinetic parameters by uncertainty in sampling time (± 5 min to ± 30 min on SD scale) using the accurate (grey) or planned (red) sampling times. The different scale size for each chart should be noted. For an explanation of the abbreviations, see **Figure S3**.


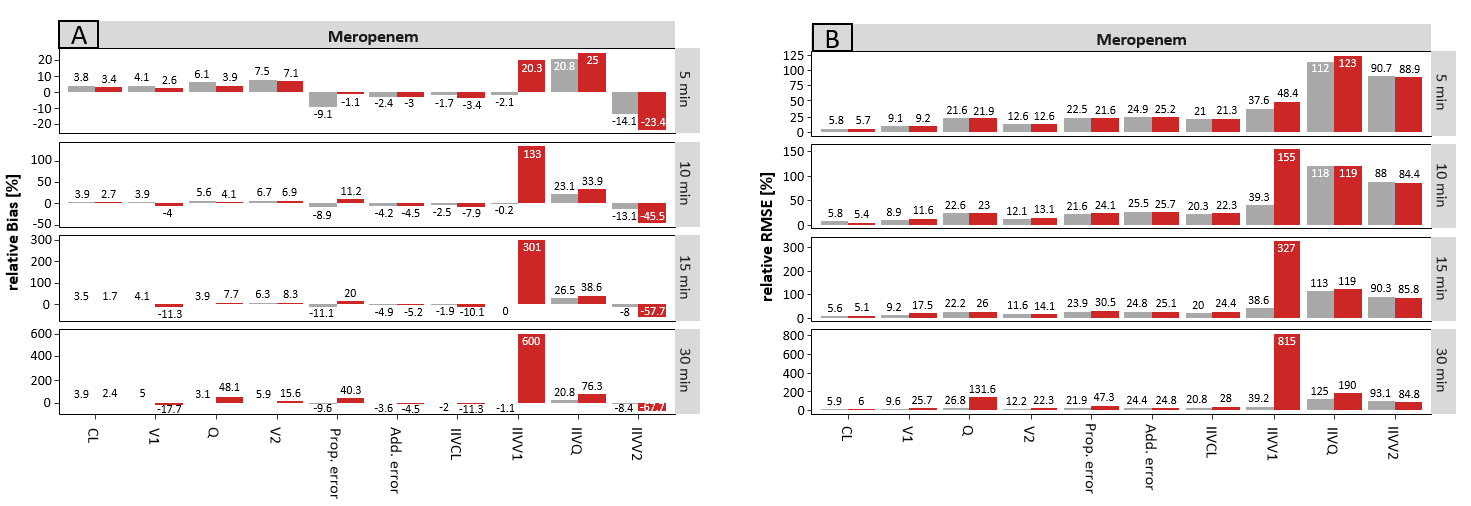


**Supplementary Figure S6**. Impact of sparse sampling. Accuracy (rBias) **(A)** and precision (rRMSE) **(B)** for meropenem population pharmacokinetic parameters by uncertainty in infusion rate (± 5 min to ± 30 min on SD scale) using the accurate (grey) or planned (red) infusion times. The different scale size for each chart should be noted. For an explanation of the abbreviations, see **Figure S2**.


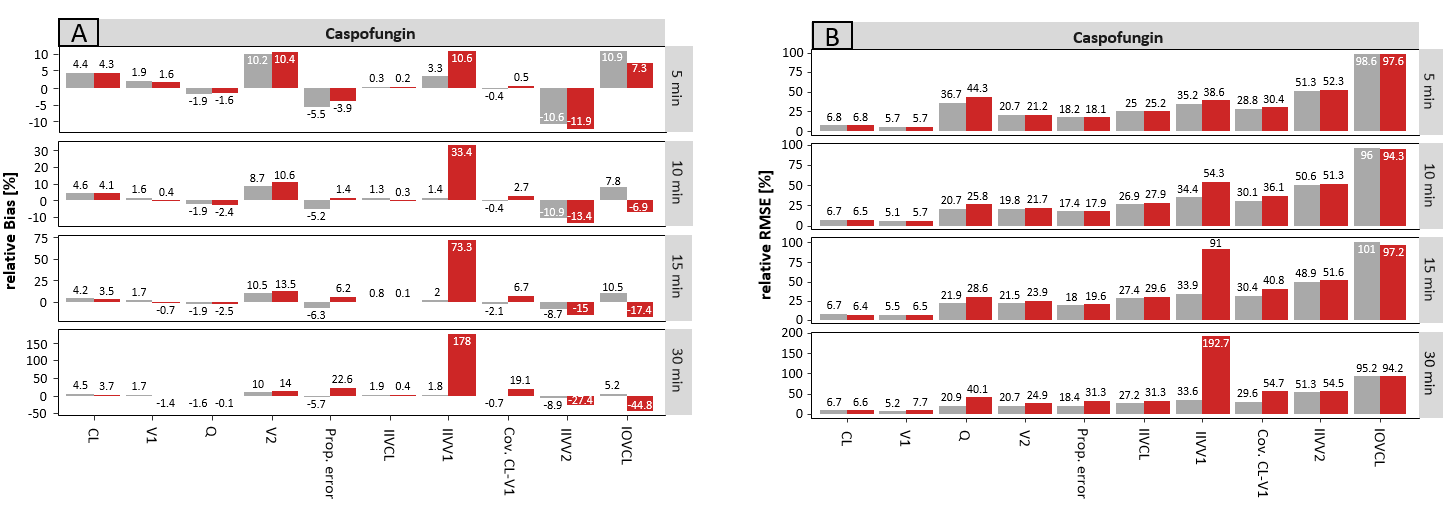


**Supplementary Figure S7.** Impact of sparse sampling. Accuracy (rBias) **(A)** and precision (rRMSE) **(B)** for caspofungin population pharmacokinetic parameters by uncertainty in infusion rate (± 5 min to ± 30 min on SD scale) using the accurate (grey) or planned (red) infusion times. The different scale size for each chart should be noted. For an explanation of the abbreviations, see **Figure S3**.


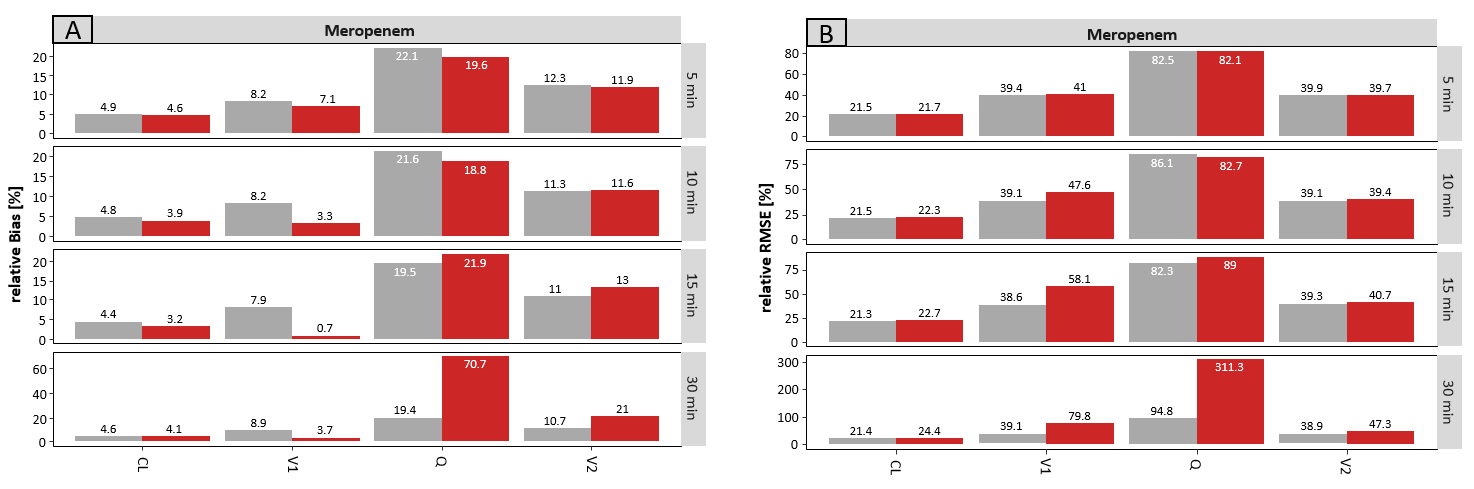


**Supplementary Figure S8.** Impact of sparse sampling. Accuracy (rBias) **(A)** and precision (rRMSE) **(B)** for meropenem individual pharmacokinetic parameters by uncertainty in infusion rate (± 5 min to ± 30 min on SD scale) using the accurate (grey) or planned (red) infusion times. The different scale size for each chart should be noted. For an explanation of the abbreviations, see **Figure S2**.


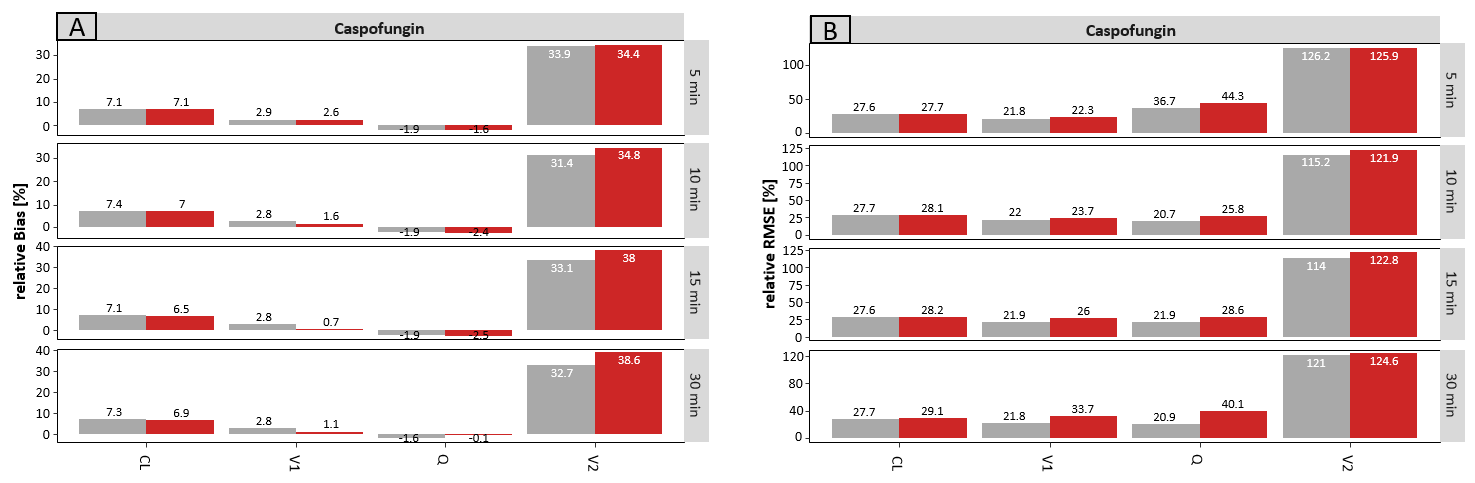


**Supplementary Figure S9.** Impact of sparse sampling. Accuracy (rBias) **(A)** and precision (rRMSE) **(B)** for caspofungin individual pharmacokinetic parameters by uncertainty in infusion rate (± 5 min to ± 30 min on SD scale) using the accurate (grey) or planned (red) infusion times. The different scale size for each chart should be noted. For an explanation of the abbreviations, see **Figure S3**.


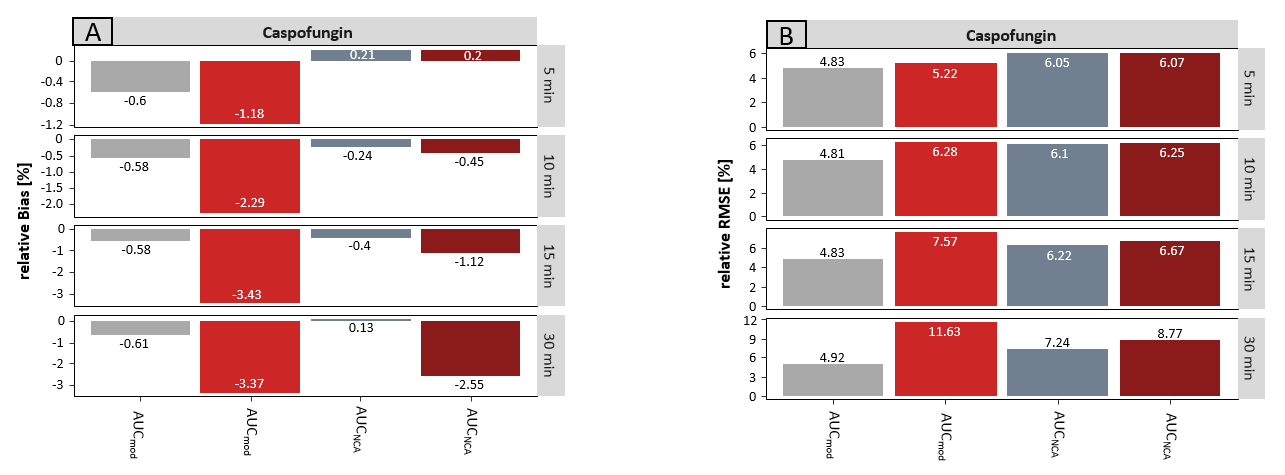


**Supplementary Figure S10.** Comparison of the model based AUC_mod_ and the AUC using the NCA (AUC_NCA_) approach. Accuracy (rBias) **(A)** and precision (rRMSE) **(B)** for caspofungin individual pharmacokinetic parameters by uncertainty in sampling time (± 5 min to ± 30 min on SD scale) using the accurate (light grey, dark grey) or planned (light red, dark red) sampling times. The different scale size for each chart should be noted.


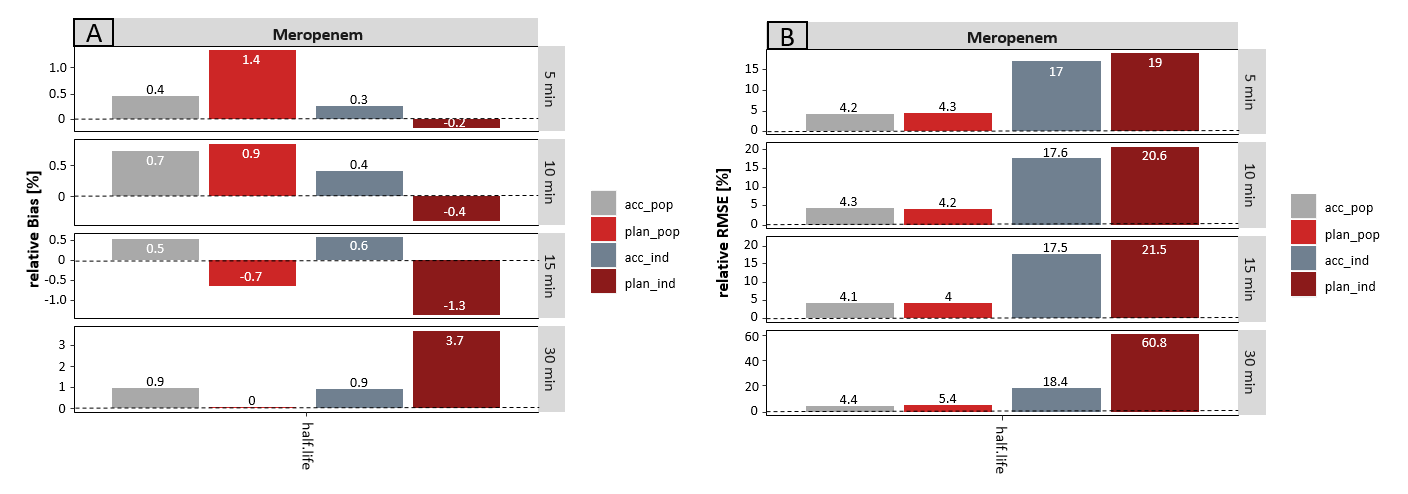


**Supplementary Figure S11.** Impact on the estimated half-life. Accuracy (rBias) **(A)** and precision (rRMSE) **(B)** for meropenem. Comparison of the population (pop) and individual (ind) pharmacokinetic parameters by uncertainty in sampling time (± 5 min to ± 30 min on SD scale) using the accurate (light grey, dark grey) or planned (light red, dark red) sampling times. The different scale size for each chart should be noted.


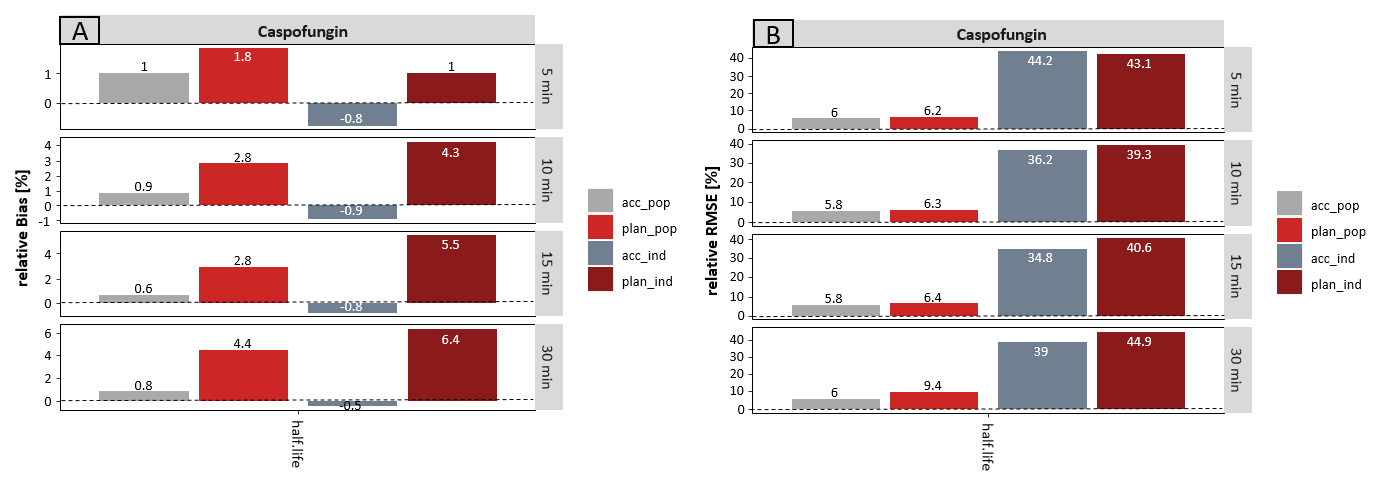


**Supplementary Figure S12.** Impact on the estimated half-life. Accuracy (rBias) **(A)** and precision (rRMSE) **(B)** for caspofungin. Comparison of the population (pop) and individual (ind) pharmacokinetic parameters by uncertainty in sampling time (± 5 min to ± 30 min on SD scale) using the accurate (light grey, dark grey) or planned (light red, dark red) sampling times. The different scale size for each chart should be noted.


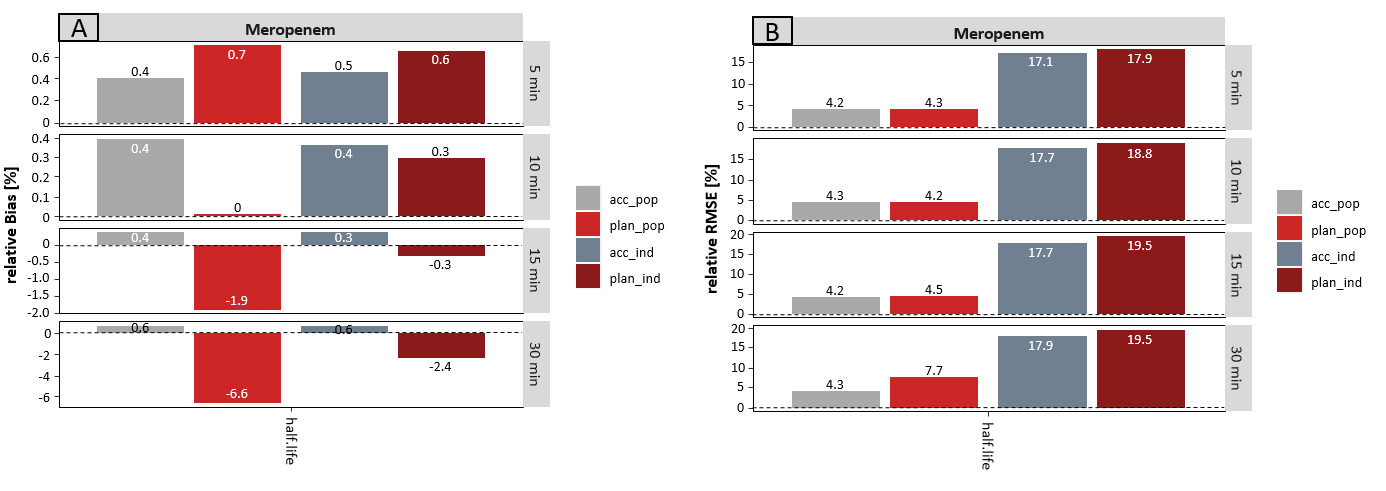


**Supplementary Figure S13.** Impact on the estimated half-life. Accuracy (rBias) **(A)** and precision (rRMSE) **(B)** for meropenem. Comparison of the population (pop) and individual (ind) pharmacokinetic parameters by uncertainty in infusion rate (± 5 min to ± 30 min on SD scale) using the accurate (light grey, dark grey) or planned (light red, dark red) infusion times. The different scale size for each chart should be noted.


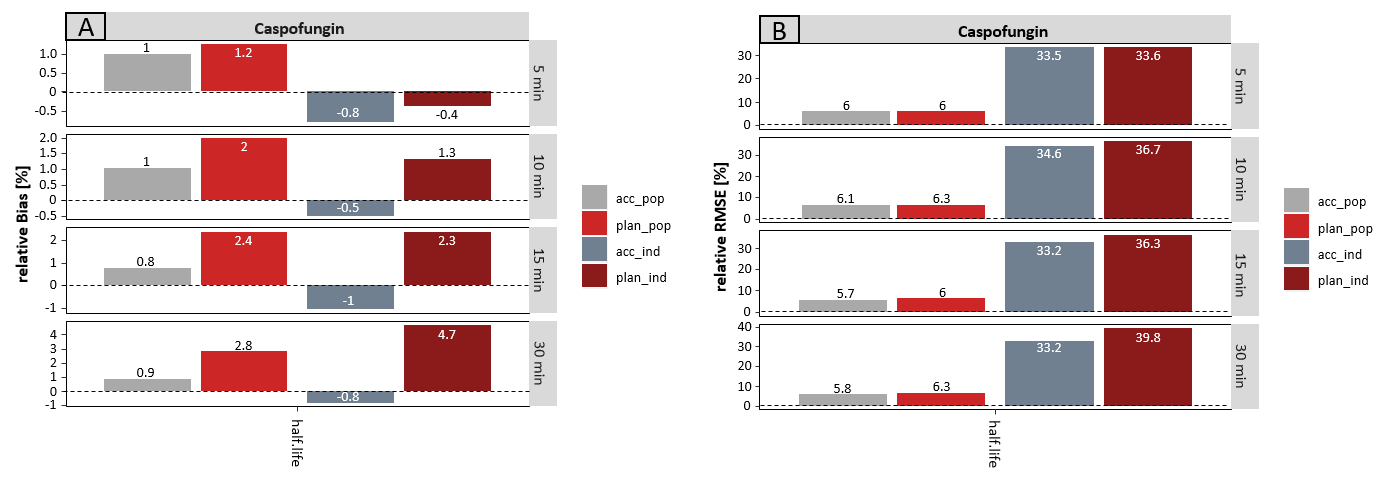


**Supplementary Figure S14.** Impact on the estimated half-life. Accuracy (rBias) **(A)** and precision (rRMSE) **(B)** for caspofungin. Comparison of the population (pop) and individual (ind) pharmacokinetic parameters by uncertainty in infusion rate (± 5 min to ± 30 min on SD scale) using the accurate (light grey, dark grey) or planned (light red, dark red) infusion times. The different scale size for each chart should be noted.
